# Supplementary material for: Systemic Investigation Identifying Salivary miR-196b as a Promising Biomarker for Early Detection of Head-Neck Cancer and Oral Precancer Lesions
Source: Diagnostics (Basel). 2021 Aug 4;11(8):1411. doi: 10.3390/diagnostics11081411 (PMC8392418; doi:10.3390/diagnostics11081411)
Supplement: Supplementary file 1 [file diagnostics-11-01411-s001.zip › diagnostics-1301621-Supplementary.pdf]

diagnostics

Supplementary Materials

Systemic Investigation Identifying Salivary miR-196b as a Promising Biomarker for Early Detection of Head-Neck Cancer and Oral Precancer Lesions

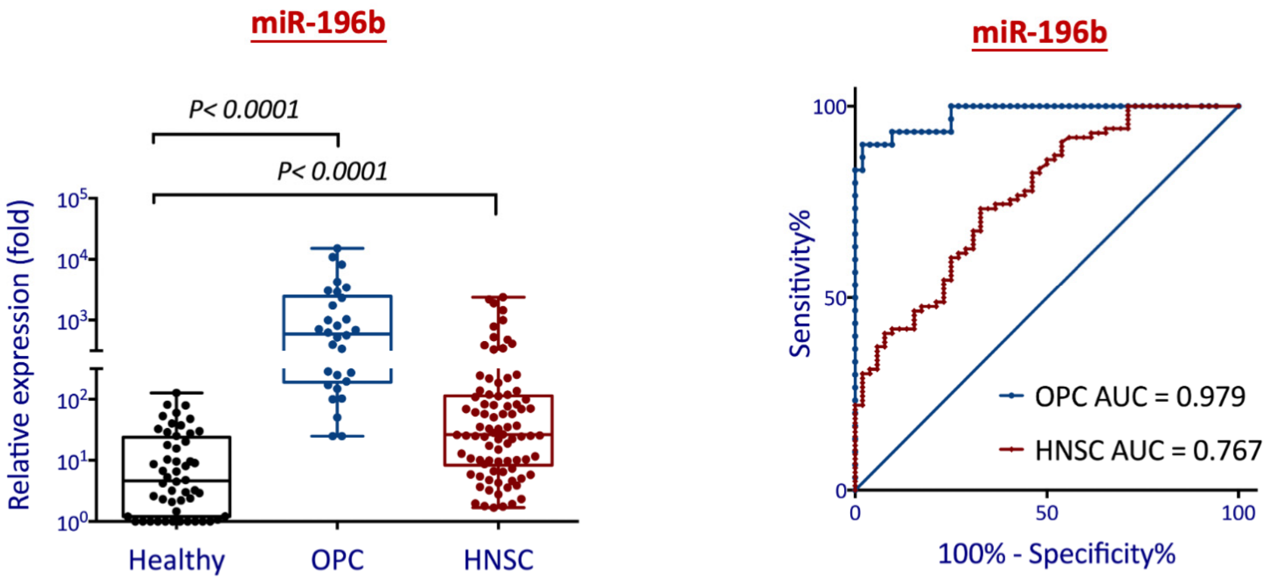

Figure S1. Elevation of miR-196b in the patients with HNSC and OPC. A total of 168 saliva samples from 52 normal individuals, 30 OPC patients, and 86 HNSC patients were recruited. Relative expression levels and the significance of miR-196b in differentiation between healthy individuals and HNSC or OPC patients. The relative expression level with (fold) was presented, after comparison to the level in OECM1 cancer cell lines (1:1000) by RT-qPCR analysis. The horizontal line across the center of the box plot represents the median value. Statistically significant differences were determined using the Mann–Whitney U test. The performance of receiver operating characteristic (ROC) curves and the area under the curve (AUC) of the ROC were shown.
